# Predictive value of cerebrovascular time constant for delayed cerebral ischemia after aneurysmal subarachnoid hemorrhage

**DOI:** 10.1177/0271678X241228512

**Published:** 2024-01-31

**Authors:** Agnieszka Uryga, Magdalena Kasprowicz, Karol Budohoski, Nathalie Nasr, Marek Czosnyka

**Affiliations:** 1 Department of Biomedical Engineering, Faculty of Fundamental Problems of Technology, Wroclaw University of Science and Technology, Wroclaw, Poland; 2 Department of Neurosurgery, Clinical Neurosciences Center, University of Utah, Salt Lake City, Utah, USA; 3 Department of Neurosurgery, University of Utah, Salt Lake City, Utah, USA; 4 Department of Neurology, Poitiers University Hospital, Laboratoire de Neurosciences Expérimentales et Cliniques, University of Poitiers, Poitiers, France; 5 Brain Physics Laboratory, Division of Neurosurgery, Department of Clinical Neurosciences, Addenbrooke’s Hospital, University of Cambridge, Cambridge, UK

**Keywords:** Cerebrovascular regulation, delayed ischemic deficit, cerebral blood flow velocity, subarachnoid haemorrhage, transcranial Doppler

## Abstract

Time constant of the cerebral arterial bed (τ) is a transcranial Doppler (TCD) based metric that is expected to quantify the transit time of red blood cells from the insonation point to the arteriole-capillary boundary during a cardiac cycle. This study aims to assess the potential of τ as an early predictor of delayed cerebral ischemia (DCI). Consecutive patients (56 ± 15 years) treated for aneurysmal subarachnoid haemorrhage were included in the study. τ was assessed through a modelling approach that involved simultaneous recordings of arterial blood pressure and cerebral blood flow velocity (CBFV) from TCD's first recordings. 71 patients were included. 17 patients experienced DCI. τ was significantly shorter in patients who later developed DCI: 187 ± 64 ms vs. 249 ± 184 ms; p = 0.040 with moderate effect size (r_G_ = 0.24). Logistic regression showed that there was a significant association between increased CBFV, shortened τ, and the development of DCI (χ^2^ = 11.54; p = 0.003) with AUC for the model 0.75. Patients who had both shortened τ and increased CBFV were 20 times more likely to develop DCI (OR = 20.4 (2.2–187.7)). Our results suggest that early alterations in τ are associated with DCI after aSAH. The highest performance of the model including both CBFV and τ may suggest the importance of both macrovascular and microvascular changes assessment.

## Introduction

Aneurysmal subarachnoid haemorrhage (aSAH) is a cerebrovascular disease characterised by high morbidity and mortality rates.^1^ One of the most severe complications after aSAH is delayed cerebral ischemia (DCI) which occurs in surviving patients after the initial haemorrhage. DCI impacts up to 30% of patients, leading to motor deficits, cognitive impairment, and a diminished quality of life in the majority of survivors.^2^ Cerebral vasospasm (CV) has long been considered the primary cause of DCI, as it can lead to a significant decrease in cerebral blood flow.^3^

Transcranial Doppler (TCD) ultrasonography has a precise, noninvasive, and efficient diagnostic tool for identifying cerebral vasospasm after aSAH.^4^ However, a solely increased cerebral blood flow velocity (CBFV) may not necessarily imply arterial narrowing, as both increasing flow and reduced vessel diameter may lead to high flow velocities.^5^ Lindegaard proposed to enhance the diagnostic accuracy of TCD for cerebral vasospasm (CV) by introducing the ratio between flow velocities in the middle cerebral artery (MCA) or anterior cerebral artery (ACA) and the internal carotid artery (ICA) as an additional criterion (Lindegaard ratio; LR).^6^ Although the LR is widely used, recent studies suggest that LR did not yield a substantial enhancement in the predictive value of TCD for the diagnosis of vasospasm^7^ and that it does not aid in the prediction of delayed neurologic deficits.^8^

More recent studies have shown that although severe CV can decrease perfusion, it may not result in DCI,^9–11^ and relying solely on CV detection using TCD is inadequate for accurately predicting the occurrence of DCI. It has been observed that a significant proportion of patients who experience DCI do not exhibit CV as detected by TCD, or vice versa.^9,10,12^ Also, cerebral infarction correlates with the territory of angiographic vasospasm in only 25% to 81% of SAH patients.^13^ Recently, a combination of vasospasm preceded by autoregulation failure was suggested as an important mechanism responsible for DCI.^14^

A study by Carrera et al.^15^ examining the relationship between TCD findings and DCI, based on an analysis of nearly two thousand TCD recordings, revealed that an elevated mean CBFV mildly increases the risk of DCI following aSAH. Intriguingly, almost 40% of patients who developed DCI did not reach a mean CBFV exceeding 120 cm/s during the monitoring period.^15^ These findings underscore the limited sensitivity of TCD recordings as a standalone predictor of DCI.

Microcirculatory disturbances have been hypothesised to play a significant role in the development of DCI after aSAH.^16,17^ To better understand cerebral hemodynamics, a novel ultrasound-based index called the time constant of cerebral arterial bed (τ) has emerged as a promising tool. τ quantifies the transit time of red blood cells from the point of insonation to the boundary between arterioles and capillaries during a cardiac cycle (see Supplementary Figure 1). In contrast to TCD, which enables the recording of the changes in CBFV in the main cerebral arteries associated with the degree of lumen narrowing, τ results from assessing both compliance of major cerebral arteries and resistance of small regulatory vessels.^18,19^

The time constant of the cerebral arterial bed is a product of two parameters: cerebrovascular resistance (CVR, defined as the mean arterial blood pressure divided by the product of the mean cerebral blood flow velocity and an unknown cross-sectional area of the vessel) and cerebral arterial compliance (C_a_, defined as the ratio between the pulse amplitude of cerebral arterial blood volume multiplied by an unknown cross-sectional area of the vessel to the pulse amplitude of arterial blood pressure). Consequently, by eliminating the common cross-sectional area factor in both the denominator and numerator of this formula τ remains independent of the insonated vessel radius and is expressed in physiologically meaningful units (milliseconds).^18,20^

τ is near-bed parameter, as its estimation requires continuous monitoring of pulse waveform of arterial blood pressure (ABP) and CBFV (see Supplementary Figure 2). Several studies have demonstrated that in patients with aSAH, τ undergoes significant changes during CV. These changes precede the onset of CV as determined by conventional TCD indices such as mean CBFV and Lindegaard ratio (LR).^21^ However, the potential of τ as an early predictor of DCI has not been investigated. The aim of this study was to assess the association between τ, derived from the initial TCD recording conducted within the first five days after aSAH, and later occurrence of DCI.

## Materials and methods

### Ethical approval

The study conforms to the Declaration of Helsinki, and the research protocol was approved by the Research Ethics Committee of Addenbrooke's Hospital (Protocol 29 LREC: 97/291). All patients or their next-of-kin were required to sign a written consent before entering the study. Data were fully anonymised, and there were no data protection issues involved.

### Study population

The study is a retrospective analysis of data collected at the Division of Neurosurgery of Addenbrooke's Hospital in Cambridge, UK between June 2010 and January 2012 from consecutive patients aged 18 years or older diagnosed with aSAH who had undergone multimodal monitoring, including ABP and CBFV within the first five days after aSAH. Patients who had poor-quality signals were excluded. The flow chart is presented in Supplementary Figure 3. This analysis is reported according to the Guidelines for Strengthening the Reporting of Observational Studies in Epidemiology (STROBE) Statement (Supplementary materials).

Patients received treatment in accordance with current guidelines for aSAH management, including cardiopulmonary support if needed, maintenance of adequate fluid volume to prevent hypovolemia, administration of oral nimodipine at a dosage of 60 mg every four hours, and external ventricular drainage in case of acute hydrocephalus.^22–24^ Surgical clipping or endovascular embolization was performed to treat the ruptured aneurysm.

### Endpoints and definitions

The primary endpoint was the occurrence of DCI within 21 days after SAH. DCI was defined as either focal neurological impairment lasting for a minimum of one hour or cerebral infarction attributable to secondary ischemia after the exclusion of other causes of cerebral infarction.^12^ Patients who were discharged before completing the 21-day study period were considered as not having DCI, unless they were readmitted for new neurological symptoms. Patients who died and had DCI within the first 21 days were classified as having DCI.^14^ Cerebral hemodynamics assessment was based on the first available transcranial Doppler (TCD) recording, performed between the day of the ictus (onset of SAH) and the fifth day thereafter by a single clinician (K.B.). This time frame was chosen based on evidence indicating that cerebral hemodynamics impairment occurring within the initial five days can predict DCI.^14,25^ CV was defined as vasospasm of the middle cerebral artery (MCA) diagnosed with TCD using mean CBFV in the MCA exceeding 120 cm/s accompanied by a Lindegaard ratio of above 3.0 or when digital subtraction angiography (DSA) was performed, as geometrical narrowing of cerebral arteries. Routine postoperative CT and DSA were not performed. In the subgroup of 20 patients who had both DSA and TCD, there was no discrepancy in the diagnosis of CV for any patients. In cases were CV was unilateral, the recording from the side with CV was considered to be the one on ipsilateral side, while the opposite side was defined as contralateral. In case of bilateral CV, an average of CBFV measurements from both sides was considered. In case CV was not present, the side of aneurysm was considered as ipsilateral side. In patients with no CV and a midline aneurysm an average of CBFV from both sides was used.

The secondary outcome was assessed at discharge from the hospital using the modified Rankin Scale (mRS). A good outcome was defined as 0–2 on the mRS and poor as 3–6 on the mRS.^26^

### Signal monitoring

Measurement of ABP was obtained invasively using a pressure transducer and a pressure monitoring kit (Baxter Healthcare, Deerfield, IL, USA) in the radial artery. CBFV was measured in the MCA through the temporal window at a depth of 45–60 mm using a TCD kit (Doppler-Box™, DWL Compumedics®, Singen, Germany) with a head positioning device (Lam Rack, DWL Compumedics). Data were digitally captured with a sampling rate of 200 Hz on bedside laptops using Intensive Care Monitoring (ICM+) software (Cambridge Enterprise Ltd, Cambridge, UK). The analysis was made based on the earliest recording of CBFV performed for each patient, if performed within the first 5 days to assess the early warning potential for DCI prediction. The median ± interquartile range (IQR) of the CBFV recording time was 68 (52–78) minutes.

### Data preprocessing

Artefacts in the recorded signals were identified through visual inspection and manually marked. Segments containing artefacts were removed from the raw signals. Then the recorded signals were processed with ICM+ software. Average values were computed from selected segments of the data considered representative. The spectral amplitude of the signals was determined using Fast Fourier Transform (FFT) within the frequency range of 0.66–3.0 Hz.

The cerebral arterial time constant (τ) was derived from a simplified cerebral circulation model,^27^ which has been described in previous publications,^28,29^ Calculation of τ requires continuous monitoring of pulse waveforms of two signals: ABP and TCD-derived CBFV. In Supplementary material we described the methodology for τ estimation. τ was defined as the product of compliance of large cerebral arteries (C_a_) and resistance of small regulatory vessels (CVR) during a cardiac cycle^21^:

(1) $$\text{τ}\;\text{=}\;{\text{C}}_{a}\cdot \mathrm{CVR}\;[ms]$$

### Statistics

Normality of data distribution was evaluated using the Shapiro-Wilk's test. Since the hypothesis of normality was rejected for most of the analysed variables, non-parametric methods were employed for further analysis. The differences in median values between groups were assessed using the U Mann-Whitney test, and the effect size was estimated using the Glass rank bi-serial correlation coefficient (r_G_). The relationship between two physiological or hemodynamic parameters was examined using the non-parametric Spearman correlation coefficient (r_S_). The frequency distribution of categorical variables was assessed using a contingency table, and differences were evaluated using the Pearson χ^2^ test (or Fisher exact test). Receiver operating characteristic (ROC) curves were constructed to determine the optimal cut-off values for parameters in predicting DCI, with the area under the curve (AUC) serving as the evaluation metric. Univariate logistic regression analysis was conducted to assess the effects of predictors (τ or CBFV) on DCI development. The joint model for DCI prediction incorporated CBFV and τ using cut-off values established in ROC curve analysis. The results of logistic regression were reported as odds ratios (OR) with corresponding 95% confidence intervals, and the model's performance was evaluated using the χ^2^ statistic. The level of significance was set at α = 0.05. All statistical analyses were conducted using STATISTICA 13 software (Tibco, Palo Alto, USA). The data are presented as median values with interquartile range (IQR) unless stated otherwise.

## Results

### Patients characteristics

In the study, a total of 71 patients were included (F/M:15/56). Median age was 56 years, ranging from 50 to 65 years. Out of the total included patients, 7 died, and out of these 7 patients, 3 were diagnosed with DCI. CV was detected in 31 patients. 17 patients developed DCI (Table 1). CBFV measurements were obtained in our cohort as follows: 18% at first 24 hours after aSAH, 27% on day 2 post-aSAH, 27% on day 3, 13% on day 4 and 15% on day 5 after aSAH. DCI was on average diagnosed on day 8 (range 4–12 days). No difference was found for sex or age between patients who developed DCI and those who did not develop DCI (Table 1). A comparison of physiological parameters between patients who did not develop DCI and patients who later developed DCI was presented in Table 2.

**Table 1.** Comparison of clinical characteristics between patients without delayed cerebral ischemia (no-DCI) and patients with DCI.

Parameter	TotalN = 71	DCIn = 17	no-DCIn = 54	p-value
Age, median ± IQR	56 ± 15	53 ± 15	57 ± 17	0.430
Female, n (%)	15 (21%)	2 (12%)	13 (24%)	0.234
GCS, median ± IQR	15 ± 5	14 ± 5	15 ± 4	0.487
Aneurysm				
ICA, n (%)	4 (6%)	2 (12%)	2 (4%)	0.246
MCA, n (%)	17 (24%)	3 (18%)	14 (26%)	0.366
ACoA, n (%)	21 (30%)	4 (24%)	17 (31%)	0.531
PComA, n (%)	21 (30%)	8 (47%)	13 (24%)	0.068
BA, n (%)	4 (6%)	1 (5%)	3 (5%)	0.675
Other, n (%)	4 (6%)	1 (5%)	3 (5%)	0.675
Clinical assessment				
mFisher grade, median ± IQR	3 ± 2	3	3 ± 2	0.500
Grade 1, n (%)	14 (20%)	1 (5%)	13 (24%)	0.358
Grade 2, n (%)	6 (9%)	2 (12%)	4 (7%)	
Grade 3, n (%)	33 (46%)	10 (59%)	23 (43%)	
Grade 4, n (%)	18 (25%)	4 (24%)	14 (26%)	
WFNS grade, median ± IQR	2 ± 3	2 ± 2	2 ± 3	0.315
Grade I, n (%)	23 (32%)	4 (24%)	19 (35%)	0.618
Grade II, n (%)	24 (34%)	6 (35%)	18 (33%)	
Grade III, n (%)	3 (4%)	0	3 (6%)	
Grade IV, n (%)	14 (20%)	5 (29%)	9 (17%)	
Grade V, n (%)	7 (10%)	2 (12%)	5 (9%)	
Aneurysm treatment				
Clipping, n (%)	47 (66%)	11 (65%)	36 (67%)	0.881
Coiling, n (%)^a^	27 (38%)	8 (47%)	19 (35%)	0.379
EVD	13 (18%)	3 (18%)	10 (19%)	0.845
Complications				
CV, n (%)	31 (44%)	14 (82%)	17 (31%)	**<0.001**
Re-bleeding, n (%)	6 (8%)	0	6 (11%)	0.151
Hydrocephalus, n (%)	37 (52%)	10 (59%)	27 (50%)	0.525
Outcome				
Dead, n (%)	7 (10%)	3 (18%)	4 (7%)	0.213
mRS, median ± IQR	2 ± 3	2 ± 2	2 ± 3	0.639

Bold font indicates significant difference between the two groups. ACoA: anterior communicating artery; BA: basilar artery; CV: cerebral vasospasm; EVD: external ventricular drainage; GCS: Glasgow Coma Scale; GOS: Glasgow Outcome Scale; ICA: internal carotid artery; mFisher: modified Fisher scale; MCA: middle cerebral artery; mRS: modified Rankin Scale; PComA: posterior communicating artery; WFNS grade: World Federation of Neurosurgical Societies grade.

a More than one type of procedure per patient allowed.

**Table 2.** Comparison of averaged physiological parameters and cerebral hemodynamic indices from first recording between patients without delayed cerebral ischemia (no-DCI) and patients with DCI.

Parameter	TotalN = 71	DCIn = 17	no-DCIn = 54	p-value
ABP [mm Hg]	90 (79–100)	90 (81–105)	89 (79–99)	0.290
AmpABP [mm Hg]	20 (16–25)	22 (19–29)	19 (15–24)	0.140
CBFV [cm/s]	69 (54–98)	91 (64–108)	66 (53–84)	**0.037**
AmpCBFV [cm/s]	22 (15–30)	27 (16–31)	22 (16–29)	0.144
HR [bpm]	71 (61–80)	73 (67–81)	71 (61–79)	0.585
C_a_ [cm/mm Hg]	0.18 (0.14–0.26)	0.16 (0.14–0.22)	0.19 (0.14–0.28)	0.477
CVR [mm Hg/cm/s]	1.37 (0.91–1.90)	0.93 (0.84–1.68)	1.48 (1.05–1.94)	0.111
τ [ms]	230 (163–340)	187 (163–227)	249 (166–350)	**0.040**

Data are presented as median (upper quartile-lower quartile). Bold font indicates significant differences. ABP: arterial blood pressure; AmpABP: spectral amplitude of arterial blood pressure pulse wave; CBFV: cerebral blood flow velocity; AmpCBFV: spectral amplitude of cerebral blood flow velocity pulse wave; HR: heart rate; C_a_: compliance of cerebral arterial bed; CVR: cerebrovascular resistance; τ: time constant of cerebral arterial bed.

### Prediction of DCI based on CBFV

**Figure 1.**
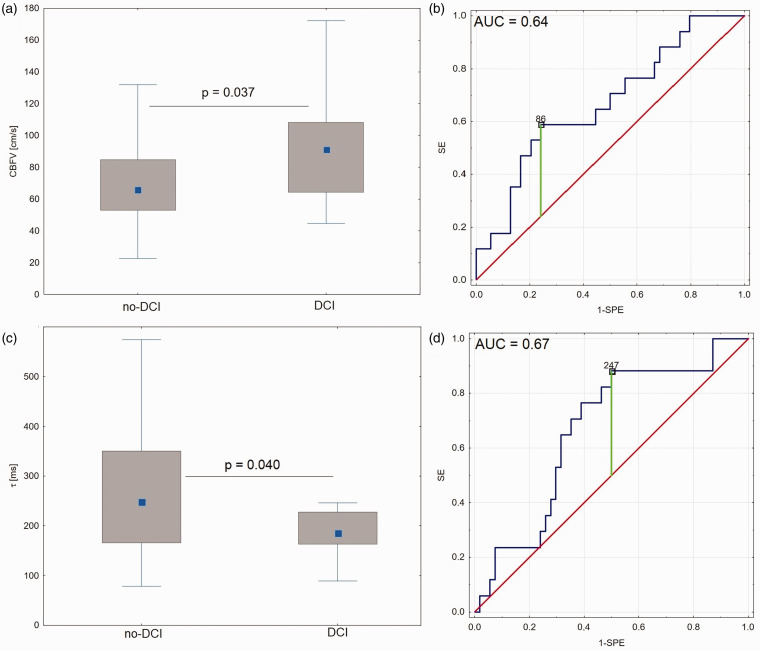
Comparison of (a) cerebral blood flow velocity (CBFV), and (c) the time constant of the cerebral arterial bed (τ), estimated from the earliest CBFV recordings performed after aneurysmal subarachnoid haemorrhage (aSAH), between patients who later developed delayed cerebral ischemia (DCI) and patients who did not later develop DCI (no-DCI). The graph presents the median values (central blue squares), interquartile ranges (grey boxes), and minimum-maximum values (ends of whiskers). (b) and (d) receiver operating curve (ROC) analysis, where DCI was predicted based on (b) CBFV, and (d) τ. Optimal cut-off values along with the area under the curve (AUC) are presented.

There was no significant difference between the ipsilateral and contralateral measurements for CBFV (p = 0.629) and τ (p = 0.909). Therefore, the results are extracted from analysis based on the averaged values of CBFV and cerebrovascular hemodynamic parameters from both sides. CBFV was higher in patients who later developed DCI as compared to patients who did not develop DCI (91 ± 44 cm/s vs. 66 ± 31 cm/s, p = 0.037). The r_G_ coefficient indicated a moderate effect size (r_G_ = 0.25), as shown in Figure 1(a). In both subgroups, CBFV values were within the normal range and did not reach the threshold for CV diagnosis, being significantly lower than 120 cm/s (p = 0.002 for patients who later developed DCI and p < 0.001 for patients who did not develop DCI). CBFV was an independent predictor of DCI (AUC = 0.64; p = 0.025; Figure 1(b)). In univariate logistic regression, elevated mean CBFV was identified as a risk factor for the development of DCI (χ^2^ = 4.78; p = 0.029; OR: 3.5 (1.1–11.1)).

### Prediction of DCI based on τ

τ was significantly shorter in patients who later developed DCI compared to patients who did not develop DCI (187 ± 64 ms vs. 249 ± 184 ms, p = 0.040). The r_G_ coefficient indicated a moderate effect size (r_G_ = 0.24, Figure 1(c)). τ was associated with later occurrence of DCI development in the ROC analysis (AUC = 0.67; p = 0.021), see Figure 1(d). Univariate logistic regression showed shortened τ to be a significant risk factor for the development of DCI (χ^2^ = 8.85; p = 0.012 with OR: 7.5 (1.6–36.0)).

### Association between increased CBFV, shortened τ, and development of DCI

**Figure 2.**
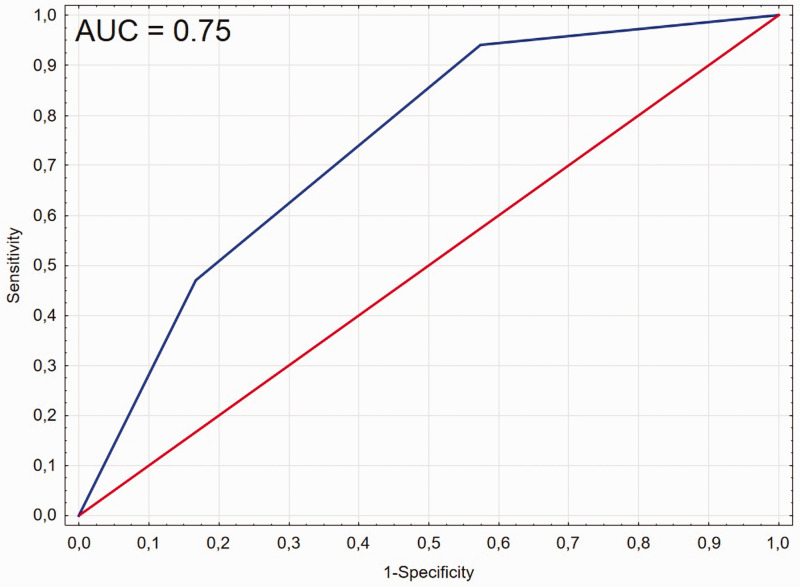
Receiver operating curve (ROC) analysis for the model, where delayed cerebral ischemia (DCI) is predicted based on the combined parameters: increased value of cerebral blood flow velocity (CBFV > 86 cm/s) and shortened the time constant of the cerebral arterial bed (τ < 247 ms).

In logistic regression, there was a significant association between increased CBFV, shortened τ, and the development of DCI (χ^2^ = 11.54; p = 0.003) with AUC for the model of 0.75 (Figure 2). Among patients with CBFV and τ within the cut-off range (τ> = 247 ms and CBFV < 86 cm/s) only 1/24 (4%) had DCI. Among patients with one of the parameters, over the range (CBFV or τ) 8/30 (27%) developed DCI. In patients with increased CBFV and decreased τ, 8/17 (47%) had DCI. Patients who had both shortened τ and increased CBFV were 20 times more likely to develop DCI (OR = 20.4 (2.2–187.7)), see Table 3.

**Table 3.** Logistic regression model of cerebral blood flow velocity (CBFV), time constant of cerebral arterial bed (τ) and its interaction to predict delayed cerebral ischemia (DCI).

	n (% of row)		OR	95% CI	p-value	AUC
	DCI	no-DCI				
CBFV						
<86 cm/s (normal)	8 (16%)	41 (84%)	1			0.64
>= 86 cm/s (abnormal)	9 (41%)	13 (59%)	3.5	1.1–11.1	**0.029**	
τ						
> = 247 ms (normal)	2 (7%)	27 (93%)	1			0.67
<247 ms (abnormal)	15 (36%)	27 (64%)	7.5	1.6–36.0	**0.012**	
τ and CBFV						
Class 1 (both normal)	1 (4%)	23 (96%)	1			0.75
Class 2 (one abnormal)	8 (27%)	22 (73%)	8.4	0.9–72.5	0.054	
Class 3 (both abnormal)	8 (47%)	9 (53%)	20.4	2.2–187.7	**0.008**	

AUC: area under the curve for receiver operative characteristic (ROC); CI: confidence interval; Class 1 (both normal): CBFV < 86 cm/s (normal) and τ > = 247 ms (normal); Class 2 (one abnormal): CBFV > = 86 cm/s (abnormal) or τ < 247 ms (abnormal); Class 3 (both abnormal): CBFV > 86 cm/s (abnormal) and τ < 247 ms (abnormal); the range determined by ROC curve threshold values; OR: odds ratio.

### Association between τ and CBFV and outcome

In the total group, 27 patients (38%) had poor outcome. Patients with poor outcome had shorter τ than ones with good outcome (187 ± 97 ms vs. 259 ± 162 ms, p = 0.035). No significant difference in CBFV was observed between these two groups. A comparison of physiological parameters between patients with good and poor outcomes is presented in Table 4.

**Table 4.** Comparison of averaged physiological parameters and cerebral hemodynamic indices from first recording between patients with poor and good outcome. Data are presented as median (upper quartile-lower quartile).

Parameter	TotalN = 71	Poor outcomen = 27	Good outcomen = 44	p-value
ABP [mm Hg]	90 (79–100)	91 (81–104)	87 (79–100)	0.328
AmpABP [mm Hg]	20 (16–25)	23 (17–27)	19 (15–23)	0.159
CBFV [cm/s]	69 (54–98)	69 (50–103)	67 (55–89)	0.957
AmpCBFV [cm/s]	22 (15–30)	22 (12–31)	23 (17–29)	0.606
HR [bpm]	71 (61–80)	75 (68–84)	69 (61–76)	**0.039**
C_a_ [cm/mm Hg]	0.18 (0.14–0.26)	0.16 (0.10–0.22)	0.19 (0.14–0.30)	0.063
CVR [mm Hg/cm/s]	1.37 (0.91–1.90)	1.55 (0.88–2.02)	1.33 (0.90–1.78)	0.488
τ [ms]	230 (163–340)	187 (149–246)	259 (187–349)	**0.035**

Bold font indicates significant differences. ABP: arterial blood pressure; AmpABP: spectral amplitude of arterial blood pressure pulse wave; CBFV: cerebral blood flow velocity; AmpCBFV: spectral amplitude of cerebral blood flow velocity pulse wave; HR: heart rate; C_a_: compliance of cerebral arterial bed; CVR: cerebrovascular resistance; τ: time constant of cerebral arterial bed.

## Discussion

In this study, we assessed τ, a parameter that reflects the transit time of blood volume through the cerebrovascular bed from the insonation point of the trunk of the MCA to the arteriole-capillary boundary, during a cardiac cycle. In our study, τ considered together with mean CBFV in the MCA enhanced the ability to predict DCI following aSAH. We observed a significant association between early decrease in τ, increase in mean CBFV and subsequent development of DCI following aSAH. The model incorporating both τ and CBFV parameters exhibited the highest performance among binary classification models. Consequently, its outperformed models that rely exclusively on either CBFV or τ, enabling more accurate prediction of prognosis.

Several mechanisms have been proposed to explain the pathophysiology of DCI in aSAH. They encompass changes in the lumen of basal cerebral arteries in the large arteries of the Circle of Willis, referred to as macrovascular cerebral circulation changes, microcirculatory dysfunction as failure of cerebral autoregulation,^14^ inverted haemodynamic response to depolarisations and cortical spreading depressions.^30–34^ The reliance on the DCI prognosis based on only one disturbance (micro- or macrovascular) seems insufficient.

The incidence of clinically symptomatic CV is reported in about 30% of aSAH patients.^35,36^ The study of Dankbaar has shown that the degree of vasospasm is related to a decrease in cerebral perfusion pressure and the occurrence of DCI.^37^ However, they also noted that almost half of the patients with severe vasospasm do not experience DCI. This study observation was confirmed in many other studies in aSAH cohort of patients.^9,10,36,38–40^ Moreover, in approximately 25% of the patient, the localization of secondary brain ischemia following aSAH does not correspond to the territory of the spastic artery,^40,41^ suggesting the involvement of other contributing factors. Therefore, the decrease in perfusion caused by CV is not sufficient to cause DCI in all patients, and the presence of CV does not necessarily imply the occurrence of DCI.^37^ Budohoski et al.^14^ proposed that if cerebral autoregulation fails before geometrical narrowing of basal arteries, DCI is more likely.

TCD remains the most commonly employed imaging technique for the detection of CV. Acceleration of CBFV measured by TCD more than 120 cm/s is sensitive to the presence of angiographic vasospasm in the proximal MCA, but TCD is insensitive to spasm of the distal vasculature.^42^ The study of Carrera^15^ has shown that the sensitivity of mean CBFV more than 120 cm/s as a DCI predictor is about 60%, which means that almost 40% of patients with DCI did not reach this CBFV before manifestation of DCI. What’s more, they showed that among patients with DCI, 16% of them never had a mean CBFV of more than 120 cm/s.^15^ As an alternative approach Papaioannou et al.^43^ proposed using slow-wave of CBFV to describe a link between CV and DCI in patients suffering from SAH.

τ estimates the time for blood arrival at the arteriole-capillary border. The added value of τ as compared to CV or CBFV alone probably originates from the fact that it encompasses hemodynamic changes along the cerebrovascular bed downstream from the insonation point till the arteriole-capillary border, independently of changes in the vessels’ diameter. Our hypothesis concerning the association between τ and delayed ischemic deficit is that τ reduction may be driven by decreased compliance in this specific context due to both stiffening of more proximal parts of the cerebral vasculature hindering their further increase in volume in response to ABP on one hand, and dilation of more distal parts of arterial segments impeding more vasodilation of these segments on the other hand.^32,44^ In our study, both CBFV and τ had moderate accuracy as DCI predictors. The model that integrated both τ and CBFV parameters demonstrated the highest performance. The significant performance improvement when combining both may suggest the importance of both macrovascular and microvascular changes in prediction DCI.

Our study focused on the τ index, which provides insights into the dynamic interaction between cerebral arterial compliance and cerebrovascular resistance. Importantly, this index remains unaffected by the size of the insonated cerebral artery which makes it of particular interest in vasospasm affecting arteries of different diameters following SAH.^21^ In addition to evaluating large arterial vessels, τ provides supplementary information regarding the state of the cerebral vasculature beyond the insonation point, encompassing the downstream region up to the arteriole-capillary border.

This study has limitations. In our analysis, we used data collected from patients who were hospitalised several years ago. Nevertheless, patients’ management followed standardised protocols that were close to the recently published treatment guidelines.^22–24,45^ The limited number of patients with DCI may have contributed to the absence of an association between haemorrhage extension in the Fisher scale or severity of aSAH in World Federation of Neurosurgical Societies (WFNS) scale and the development of DCI. Because of the observational single-centre design and relatively small study population, these results need to be confirmed in a large multicentre study Importantly, in a clinical context, the τ should be analysed together with neuroimaging, electrophysiological monitoring, and metabolic biomarkers to reflect the real-time changes in brain injury haemodynamics.

## Conclusion

Our results suggest that early alterations in τ are associated with DCI after aSAH. The highest performance of the model including both CBFV and τ might suggest the importance of both macrovascular and microvascular early changes in the occurrence of DCI.

## Supplemental Material

sj-pdf-1-jcb-10.1177_0271678X241228512 - Supplemental material for Predictive value of cerebrovascular time constant for delayed cerebral ischemia after aneurysmal subarachnoid hemorrhage Supplemental material, sj-pdf-1-jcb-10.1177_0271678X241228512 for Predictive value of cerebrovascular time constant for delayed cerebral ischemia after aneurysmal subarachnoid hemorrhage by Agnieszka Uryga, Magdalena Kasprowicz, Karol Budohoski, Nathalie Nasr and Marek Czosnyka Prof in Journal of Cerebral Blood Flow & Metabolism

sj-pdf-2-jcb-10.1177_0271678X241228512 - Supplemental material for Predictive value of cerebrovascular time constant for delayed cerebral ischemia after aneurysmal subarachnoid hemorrhage Supplemental material, sj-pdf-2-jcb-10.1177_0271678X241228512 for Predictive value of cerebrovascular time constant for delayed cerebral ischemia after aneurysmal subarachnoid hemorrhage by Agnieszka Uryga, Magdalena Kasprowicz, Karol Budohoski, Nathalie Nasr and Marek Czosnyka Prof in Journal of Cerebral Blood Flow & Metabolism

sj-pdf-3-jcb-10.1177_0271678X241228512 - Supplemental material for Predictive value of cerebrovascular time constant for delayed cerebral ischemia after aneurysmal subarachnoid hemorrhage Supplemental material, sj-pdf-3-jcb-10.1177_0271678X241228512 for Predictive value of cerebrovascular time constant for delayed cerebral ischemia after aneurysmal subarachnoid hemorrhage by Agnieszka Uryga, Magdalena Kasprowicz, Karol Budohoski, Nathalie Nasr and Marek Czosnyka Prof in Journal of Cerebral Blood Flow & Metabolism

sj-pdf-4-jcb-10.1177_0271678X241228512 - Supplemental material for Predictive value of cerebrovascular time constant for delayed cerebral ischemia after aneurysmal subarachnoid hemorrhage Supplemental material, sj-pdf-4-jcb-10.1177_0271678X241228512 for Predictive value of cerebrovascular time constant for delayed cerebral ischemia after aneurysmal subarachnoid hemorrhage by Agnieszka Uryga, Magdalena Kasprowicz, Karol Budohoski, Nathalie Nasr and Marek Czosnyka Prof in Journal of Cerebral Blood Flow & Metabolism

sj-pdf-5-jcb-10.1177_0271678X241228512 - Supplemental material for Predictive value of cerebrovascular time constant for delayed cerebral ischemia after aneurysmal subarachnoid hemorrhage Supplemental material, sj-pdf-5-jcb-10.1177_0271678X241228512 for Predictive value of cerebrovascular time constant for delayed cerebral ischemia after aneurysmal subarachnoid hemorrhage by Agnieszka Uryga, Magdalena Kasprowicz, Karol Budohoski, Nathalie Nasr and Marek Czosnyka Prof in Journal of Cerebral Blood Flow & Metabolism
