# Supplementary material for: Predictive value of cerebrovascular time constant for delayed cerebral ischemia after aneurysmal subarachnoid hemorrhage
Source: J Cereb Blood Flow Metab. 2024 Jan 31;44(7):1208–17. doi: 10.1177/0271678X241228512 (PMC11179618; doi:10.1177/0271678X241228512)
Supplement: sj-pdf-1-jcb-10.1177_0271678X241228512 - Supplemental material for Predictive value of cerebrovascular time constant for delayed cerebral ischemia after aneurysmal subarachnoid hemorrhage [file sj-pdf-1-jcb-10.1177_0271678X241228512.pdf]

## Supplementary materials

### *Changes in cerebral arterial blood volume*

Changes in cerebral blood volume ($\Delta C_a BV$) during a cardiac cycle are calculated as an integral of the difference between pulsatile arterial inflow and venous outflow of cerebral blood [1]. As venous outflow is relatively low pulsatile, it is expressed as the averaged arterial inflow. According to the continuous flow forward model (CFF), $\Delta C_a BV$ is expressed under the assumption of a constant cross-sectional area of the insonated vessel ($S_a$) and utilizing TCD as follows [2]:

$$\Delta C_a BV = \sum_{i=1}^{n} (CBFV(i) - CBFV_m)\Delta t \quad [cm] \tag{1}$$

where: n – following number of samples, $\Delta t$ is the time interval between two subsequent samples, CBFV(i) are the samples of the cerebral blood flow velocity (CBFV), and $CBFV_m$ is the moving–average of CBFV from the window including several previous heart cycles (a 6–second window was applied). $\Delta C_a BV$ was normalized for further calculation (divided into the unknown $S_a$ [$cm^2$]), resulting in expression in [cm] [3].

### *Cerebrovascular resistance*

Cerebrovascular resistance (CVR) is a resistance of small cerebral arteries and arterioles. It is defined as a ratio between mean cerebral perfusion pressure (CPP) and mean cerebral blood flow (CBF). Under the assumption of relatively low intracranial pressure (ICP), CPP was approximated by mean arterial blood pressure ($ABP_m$), and CBF was approximated by $CBFV_m$:

$$CVR = \frac{ABP_m}{CBFV_m} \quad \left[\frac{mmHg}{cm/s}\right] \tag{2}$$

### *Compliance of the cerebral arterial bed*

Compliance of cerebral arterial bed ($C_a$) is estimated as the ratio between the amplitude of $\Delta C_a BV$ ($Amp\Delta C_a BV$) and amplitude of ABP (AmpABP) as follows:

$$C_a = \frac{Amp\Delta C_a BV}{AmpABP} \quad \left[\frac{cm}{mm\,Hg}\right] \tag{3}$$

The amplitudes of fundamental components (first harmonic) of ABP and ΔCaBV were calculated using Fourier Transform.

## Limitations of continuous flow forward model

We assumed that systemic ABP waveforms from the radial artery represents cerebral perfusion pressure waveforms. It is however likely, based on the modelling study bay Zamir et al. that this assumption did not introduce significant bias [4].

References:


[1] Avezaat CJJ, van Eijndhoven JHM. The role of the pulsatile pressure variations in intracranial pressure monitoring. *Neurosurg Rev*. 1986;9:113–120.

[2] Czosnyka M, Piechnik S, Richards HK, Kirkpatrick P, Smielewski P, Pickard JD. Contribution of mathematical modelling to the interpretation of bedside tests of cerebrovascular autoregulation. *J Neurol Neurosurg Psychiatry*. 1997;63:721–31.

[3] Czosnyka M, Richards HK, Reinhard M, Steiner L a, Budohoski K, Smielewski P, Pickard JD, Kasprowicz M. Cerebrovascular time constant: dependence on cerebral perfusion pressure and end-tidal carbon dioxide concentration. *Neurol Res*. 2012;34:17–24.

[4] Zamir M, Moir ME, Klassen SA, et al. Cerebrovascular compliance within the rigid confines of the skull. Front Physiol. 2018;9:940. https ://doi.org/10.3389/fphys .2018.00940 .

**"Strengthening the Reporting of Observational Studies in Epidemiology (STROBE)" statement guidelines.**

| | Item No. | Recommendation | Page No. | Relevant text from manuscript |
|---|---|---|---|---|
| **Title and abstract** | 1 | (*a*) Indicate the study's design with a commonly used term in the title or the abstract | 1 | |
| | | (*b*) Provide in the abstract an informative and balanced summary of what was done and what was found | 2 | |
| **Introduction** | | | | |
| Background/rationale | 2 | Explain the scientific background and rationale for the investigation being reported | 3 | |
| Objectives | 3 | State specific objectives, including any prespecified hypotheses | 3-4 | |
| **Methods** | | | | |
| Study design | 4 | Present key elements of study design early in the paper | 4-5 | |
| Setting | 5 | Describe the setting, locations, and relevant dates, including periods of recruitment, exposure, follow-up, and data collection | 5 | |
| Participants | 6 | (*a*) *Cohort study*—Give the eligibility criteria, and the sources and methods of selection of participants. Describe methods of follow-up | 5 | |
| | | | | |
| Variables | 7 | Clearly define all outcomes, exposures, predictors, potential confounders, and effect modifiers. Give diagnostic criteria, if applicable | 5-6 | |
| Data sources/ measurement | 8* | For each variable of interest, give sources of data and details of methods of assessment (measurement). Describe comparability of assessment methods if there is more than one group | 5-6 | |
| Bias | 9 | Describe any efforts to address potential sources of bias | NA | |

| | | | |
|---|---|---|---|
| Study size | 10 | Explain how the study size was arrived at | 5 |
| Quantitative variables | 11 | Explain how quantitative variables were handled in the analyses. If applicable, describe which groupings were chosen and why | 6-7 |
| Statistical methods | 12 | (*a*) Describe all statistical methods, including those used to control for confounding | 6-7 |
| | | (*b*) Describe any methods used to examine subgroups and interactions | |
| | | (*c*) Explain how missing data were addressed | |
| | | | |
| | | (d) Describe any sensitivity analyses | NA |
| **Results** | | | |
| Participants | 13* | (a) Report numbers of individuals at each stage of study—eg numbers potentially eligible, examined for eligibility, confirmed eligible, included in the study, completing follow-up, and analyzed | 7 |
| | | (b) Give reasons for non-participation at each stage | |
| | | (c) Consider use of a flow diagram | |
| Descriptive data | 14* | (a) Give characteristics of study participants (eg demographic, clinical, social) and information on exposures and potential confounders | 7 |
| | | (b) Indicate number of participants with missing data for each variable of interest | |
| | | (c) *Cohort study*—Summaries follow-up time (eg, average and total amount) | |
| Outcome data | 15* | *Cohort study*—Report numbers of outcome events or summary measures over time | 7-8 |
| | | *Case-control study*—Report numbers in each exposure category, or summary measures of exposure | |
| | | *Cross-sectional study*—Report numbers of outcome events or summary measures | |
| Main results | 16 | (*a*) Give unadjusted estimates and, if applicable, confounder-adjusted estimates and their precision (eg, | 7-8 |

| | | | |
|---|---|---|---|
| | | 95% confidence interval). Make clear which confounders were adjusted for and why they were included | |
| | | (*b*) Report category boundaries when continuous variables were categorized | |
| | | (*c*) If relevant, consider translating estimates of relative risk into absolute risk for a meaningful time period | |
| Other analyses | 17 | Report other analyses done—eg analyses of subgroups and interactions, and sensitivity analyses | 7-8 |
| **Discussion** | | | |
| Key results | 18 | Summaries key results with reference to study objectives | 9 |
| Limitations | 19 | Discuss limitations of the study, taking into account sources of potential bias or imprecision. Discuss both direction and magnitude of any potential bias | 10 |
| Interpretation | 20 | Give a cautious overall interpretation of results considering objectives, limitations, multiplicity of analyses, results from similar studies, and other relevant evidence | 9-10 |
| Generalizability | 21 | Discuss the generalizability (external validity) of the study results | 10-11 |
| **Other information** | | | |
| Funding | 22 | Give the source of funding and the role of the funders for the present study and, if applicable, for the original study on which the present article is based. | 11 |

*Give information separately for cases and controls in case-control studies and, if applicable, for exposed and unexposed groups in cohort and cross-sectional studies.

**Note:** An Explanation and Elaboration article discusses each checklist item and gives methodological background and published examples of transparent reporting. The STROBE checklist is best used in conjunction with this article (freely available on the Web sites of PLoS Medicine at http://www.plosmedicine.org/, Annals of Internal Medicine at http://www.annals.org/, and Epidemiology at http://www.epidem.com/). Information on the STROBE Initiative is available at www.strobe-statement.org
