# Supplementary material for: Predictive value of cerebrovascular time constant for delayed cerebral ischemia after aneurysmal subarachnoid hemorrhage
Source: J Cereb Blood Flow Metab. 2024 Jan 31;44(7):1208–17. doi: 10.1177/0271678X241228512 (PMC11179618; doi:10.1177/0271678X241228512)
Supplement: sj-pdf-2-jcb-10.1177_0271678X241228512 - Supplemental material for Predictive value of cerebrovascular time constant for delayed cerebral ischemia after aneurysmal subarachnoid hemorrhage [file sj-pdf-2-jcb-10.1177_0271678X241228512.pdf]

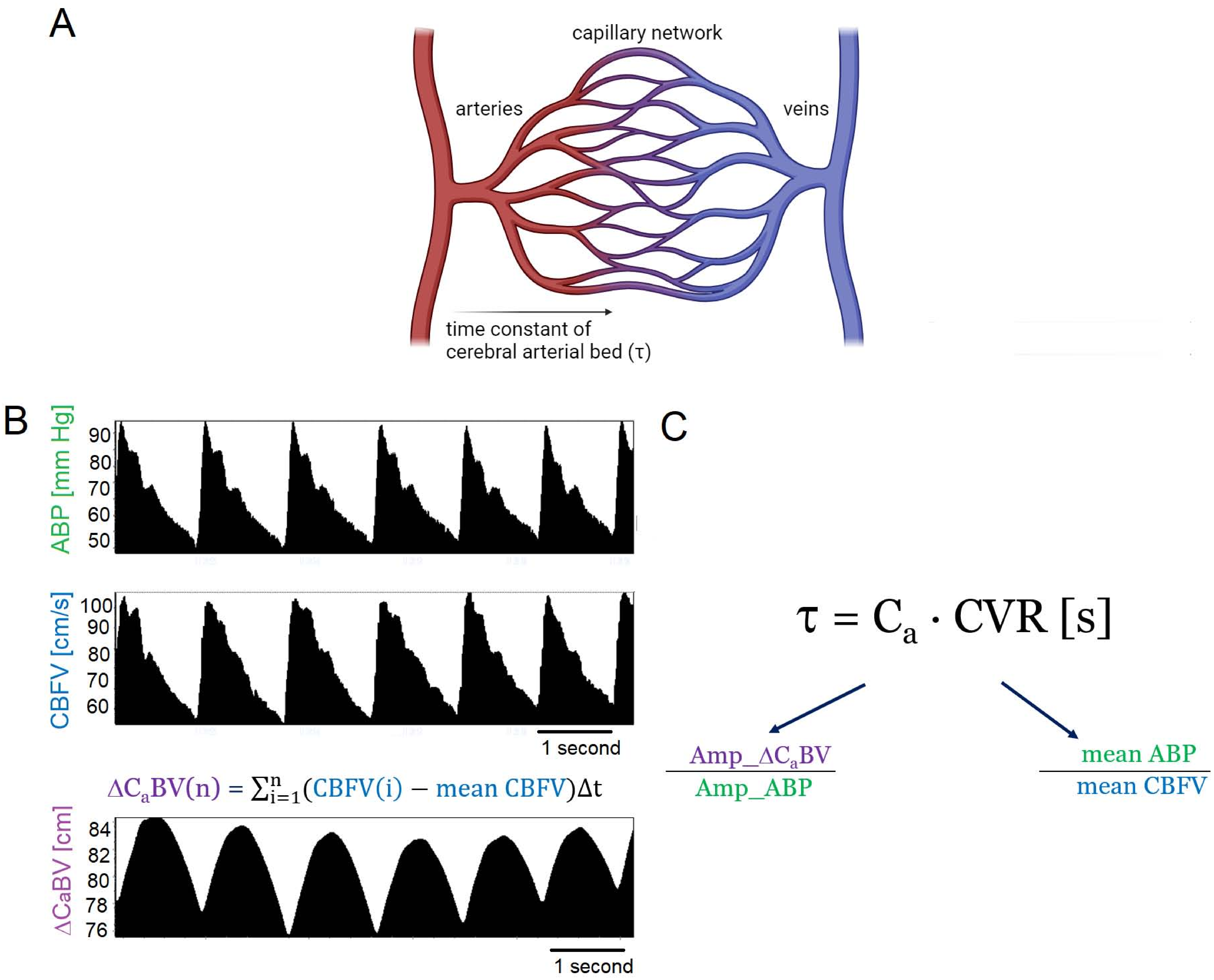
A
capillary network
arteries
veins
time constant of
cerebral arterial bed (τ)
B
ABP [mm Hg]
90
80
70
60
50
CBFV [cm/s]
100
90
80
70
60
1 second
ΔCaBV(n) = Σi=1n(CBFV(i) − mean CBFV)Δt
ΔCaBV [cm]
84
82
80
78
76
1 second
C
τ = Ca · CVR [s]
Amp_ΔCaBV / Amp_ABP
mean ABP / mean CBFV
