# Supplementary material for: Predictive value of cerebrovascular time constant for delayed cerebral ischemia after aneurysmal subarachnoid hemorrhage
Source: J Cereb Blood Flow Metab. 2024 Jan 31;44(7):1208–17. doi: 10.1177/0271678X241228512 (PMC11179618; doi:10.1177/0271678X241228512)
Supplement: sj-pdf-3-jcb-10.1177_0271678X241228512 - Supplemental material for Predictive value of cerebrovascular time constant for delayed cerebral ischemia after aneurysmal subarachnoid hemorrhage [file sj-pdf-3-jcb-10.1177_0271678X241228512.pdf]

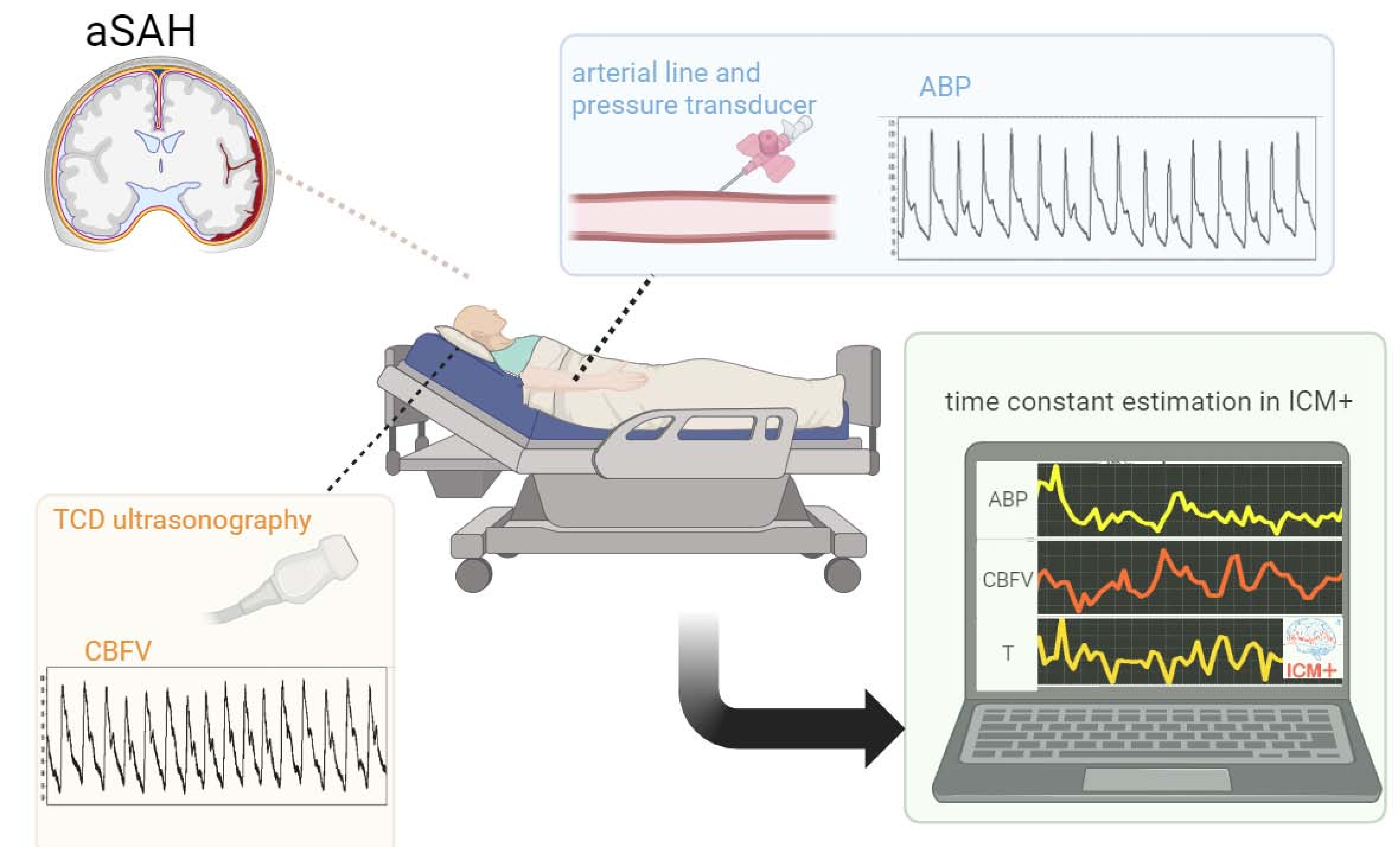
aSAH
arterial line and
pressure transducer
ABP
time constant estimation in ICM+
ABP
CBFV
T
ICM+
TCD ultrasonography
CBFV
