# Supplementary material for: Predictive value of cerebrovascular time constant for delayed cerebral ischemia after aneurysmal subarachnoid hemorrhage
Source: J Cereb Blood Flow Metab. 2024 Jan 31;44(7):1208–17. doi: 10.1177/0271678X241228512 (PMC11179618; doi:10.1177/0271678X241228512)
Supplement: sj-pdf-4-jcb-10.1177_0271678X241228512 - Supplemental material for Predictive value of cerebrovascular time constant for delayed cerebral ischemia after aneurysmal subarachnoid hemorrhage [file sj-pdf-4-jcb-10.1177_0271678X241228512.pdf]

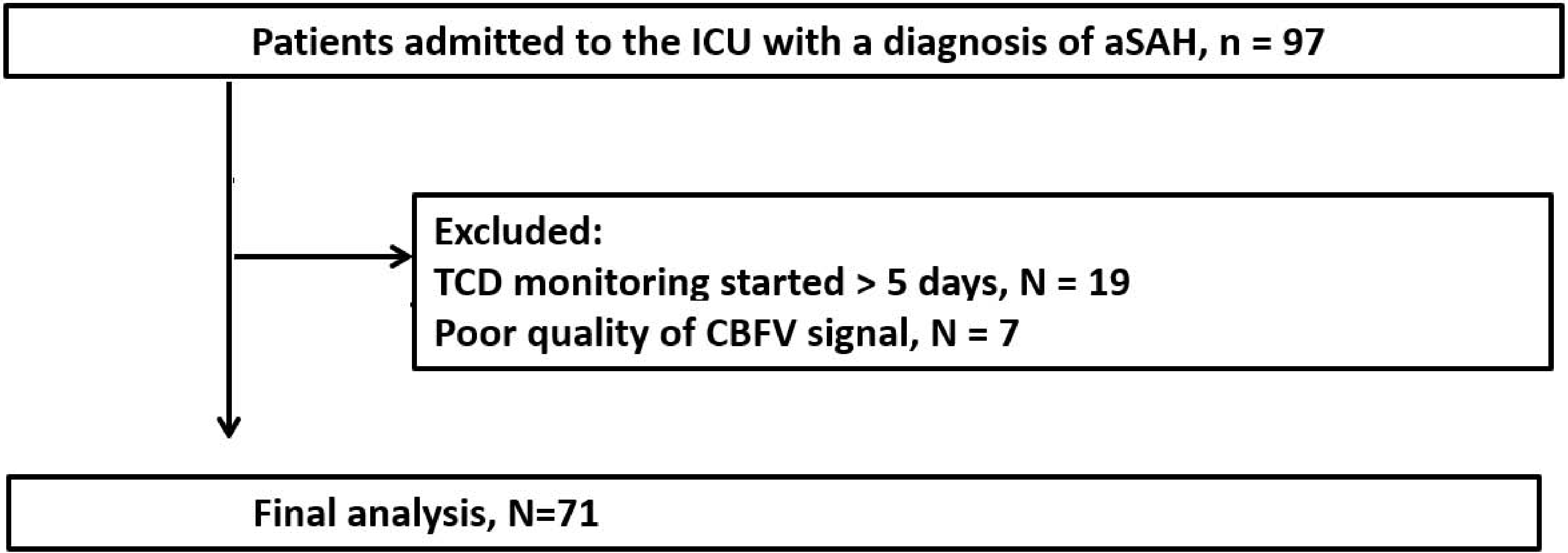
Patients admitted to the ICU with a diagnosis of aSAH, n = 97
Excluded:
TCD monitoring started > 5 days, N = 19
Poor quality of CBFV signal, N = 7
Final analysis, N=71
