# Supplementary material for: Predictive value of cerebrovascular time constant for delayed cerebral ischemia after aneurysmal subarachnoid hemorrhage
Source: J Cereb Blood Flow Metab. 2024 Jan 31;44(7):1208–17. doi: 10.1177/0271678X241228512 (PMC11179618; doi:10.1177/0271678X241228512)
Supplement: sj-pdf-5-jcb-10.1177_0271678X241228512 - Supplemental material for Predictive value of cerebrovascular time constant for delayed cerebral ischemia after aneurysmal subarachnoid hemorrhage [file sj-pdf-5-jcb-10.1177_0271678X241228512.pdf]

Supplementary Figure 1

A. The τ estimates the time for blood arrival at the arteriole-capillary border. The added value of τ originates from the fact that it encompasses hemodynamic changes along the cerebrovascular bed downstream from the insonation point till the arteriole-capillary border, these changes being independent of the vessels' diameter. **B.** Time constant of the cerebral arterial (τ) bed is estimated from continuous monitoring of arterial blood pressure (ABP) and cerebral blood flow velocity (CBFV) obtained from Transcranial Doppler ultrasonography. **C**. τ takes into account changes in cerebral arterial compliance (C_a) and cerebrovascular resistance (CVR). Abbreviations: $\Delta C_a BV$, changes in cerebral blood volume; n, following number of samples; Δt, the time interval between two subsequent samples, CBFV(i), the samples of the cerebral blood flow velocity (CBFV), $Amp\Delta C_a BV$, pulse amplitude of cerebral arterial blood volume; AmpABP, amplitude of arterial blood pressure; mean ABP, mean of arterial blood pressure; mean CBFV, mean of cerebral blood flow velocity.

Supplementary Figure 2

Estimation of the time constant of cerebral arterial bed (τ) utilises two continuous monitoring, high-frequency signals: arterial blood pressure (ABP) and transcranial Doppler (TCD) cerebral blood flow velocity (CBFV). τ is estimated in real-time based on the mathematical transformation of ABP and CBFV pulse waveforms embedded in Intensive Care Monitor (ICM+) software installed near the patient's bed mode.

Supplementary Figure 3.

Flow chart. Abbreviations: aSAH, aneurysmal subarachnoid haemorrhage; CBFV, cerebral blood flow velocity; ICU, Intensive Care Unit; TCD, transcranial Doppler
